# A neurotherapeutic approach with *Lacticaseibacillus rhamnosus* E9 on gut microbiota and intestinal barrier in MPTP-induced mouse model of Parkinson’s disease

**DOI:** 10.1038/s41598-024-65061-w

**Published:** 2024-07-04

**Authors:** Busra Aktas, Belma Aslim, Deniz Ates Ozdemir

**Affiliations:** 1 Department of Molecular Biology and Genetics, Burdur Mehmet Akif Ersoy University, Burdur, 15200 Turkey; 2 Department of Biology, Faculty of Science, Gazi University, Ankara, 06500 Turkey; 3 Department of Pathology, Faculty of Medicine, Hacettepe University, Ankara, 06230 Turkey

**Keywords:** *Lacticaseibacillus rhamnosus*, Neurotherapeutic, Parkinson’s disease, Gut microbiota, Biotechnology, Microbiology, Molecular biology, Neuroscience

## Abstract

The gut microbiota plays a crucial role in neural development and progression of neural disorders like Parkinson’s disease (PD). Probiotics have been suggested to impact neurodegenerative diseases via gut-brain axis. This study aims to investigate the therapeutic potential of *Lacticaseibacillus rhamnosus* E9, a high exopolysaccharide producer, on 1-methyl-4-phenyl-1,2,3,6-tetrahydropyridine(MPTP)-induced mouse model of PD. C57BL/6 mice subjected to MPTP were fed *L. rhamnosus* E9 for fifteen days and sacrificed after the last administration. Motor functions were determined by open-field, catalepsy, and wire-hanging tests. The ileum and the brain tissues were collected for ELISA, qPCR, and immunohistochemistry analyses. The cecum content was obtained for microbiota analysis. E9 supplementation alleviated MPTP-induced motor dysfunctions accompanied by decreased levels of striatal TH and dopamine. E9 also reduced the level of ROS in the striatum and decreased the DAT expression while increasing the DR1. Furthermore, E9 improved intestinal integrity by enhancing ZO-1 and Occludin levels and reversed the dysbiosis of the gut microbiota induced by MPTP. In conclusion, E9 supplementation improved the MPTP-induced motor deficits and neural damage as well as intestinal barrier by modulating the gut microbiota in PD mice. These findings suggest that E9 supplementation holds therapeutic potential in managing PD through the gut-brain axis.

## Introduction

Parkinson's disease (PD) is a slowly progressive neurodegenerative disease of the central nervous system with a high prevalence^1–5^. The most characteristic feature of PD is the loss of dopaminergic neurons^6–8^. Degeneration of dopamine producing neurons, which often causes motor dysfunctions such as postural instability, bradykinesia, rest tremor, and rigidity, is not yet clearly understood. Factors associated with the neural degeneration in PD include mitochondrial dysfunction, increase in free radicals, oxidative stress, and inflammatory cytokines^7,8^. Although PD is primarily characterized by motor dysfunction, a variety of non-motor symptoms such as neurocognitive impairment, sleep disturbances, leaky gut syndrome, and defecation dysfunction also have been observed in PD patients^9,10^. The most commonly reported symptom among the non-motor manifestations is constipation. More interestingly, the gut-related symptoms are experienced years before the motor symptoms^9–11^. Gastrointestinal dysfunction, hence, could be involved in the presymptomatic phase of PD.

Growing evidence suggests that the gut and the brain communicate with each other and contribute to development and progression of PD^12^. Gut microbiota has been recognized as an important mediator of the gut-brain communication affecting brain development and function^13–15^. Disruption of the microbiota, known as dysbiosis, is found to be related to development of disease status in a healthy individual^16–18^. Dysbiosis leading to leaky gut syndrome due to disruption of the intestinal integrity causes neuroinflammation and plays a key role in neurodegenerative diseases including PD. In studies with PD patients and healthy individuals, intestinal integrity has been observed to be impaired in the PD patients^17,18^. Likewise, in stool samples collected from PD patients and their matched healthy controls, gut microbiota was found to be altered in the PD patients compared to the healthy controls^19–21^. In an MPTP-induced PD mouse model, fecal transplantation from healthy mice showed that the increased levels of short-chain fatty acids were reduced, the disrupted intestinal microbiota was balanced, and the neuroinflammation was suppressed^21^. There are currently no therapeutic approaches delaying PD progression, mostly include replenishing dopamine to provide a temporary symptomatic relief for motor symptoms^22–25^. Alternative therapeutic approaches, therefore, are needed in PD treatment or prevention. Strategies targeting modification of the gut microbiota come to the fore in the treatment of diseases associated with the gut microbiota^22,23,26–30^. Probiotics are one of the diet related approaches that have been known to alter and restructure the microbial composition in the gut^31,32^. In addition to managing gut dysbiosis, they can also help maintain immune health^33–35^. In a study where healthy individuals were given a probiotic cocktail, brain functions were measured by functional magnetic resonance imaging^36^. The activity of the brain regions controlling the central processing of emotion and sensation have found to be affected by the probiotic consumption. In another study, PD patients and their matched controls were administered a probiotic cocktail and they were evaluated based on The Movement Disorders Society-Unified Parkinson's Disease Rating Scale (MDS-UPDRS)^37^. Probiotic supplement improved the MDS-UPDRS scores as well as CRP and insulin metabolism. Studies have also shown that probiotic administration to PD patients improves constipation associated symptoms^38–40^. Although probiotics have been suggested as a potential therapeutics in PD due to their ability to modulate the gut microbiota, studies that comprehensively investigate the effect of probiotics on PD with concerning the gut-brain axis are limited^41,42^.

Previously, our research group demonstrated that *L. rhamnosus* E9 produces high amount of EPS with biologic activities such as adhering capacity, anti-proliferative, and anti-inflammatory activity and characterized the EPS structurally^43–45^. Microbial EPSs involved in probiotic activity were found to have a neuroprotective effect and thought to play a role in dopaminergic system^46–52^. Taking these into account, this study aimed to assess the therapeutic potential of *L. rhamnosus* E9 in mitigating dopaminergic neuron degeneration, oxidative stress in the striatum, and motor dysfunctions in an MPTP-induced mouse model of PD. Furthermore, considering the bidirectional communication between the gut and the brain, its impact on intestinal integrity and the gut microbiota was investigated.

## Materials and methods

### Bacterial strains

A previously described *Lacticaseibacillus rhamnosus* strain isolated from healthy infant feces (*L. rhamnosus* E9) was used in this study^43,53^. *L. rhamnosus* E9 was maintained at − 30 °C in MRS broth (Merck, Germany) with 10% (v/v) glycerol. Working cultures were prepared from frozen stocks by two sequential transfers in MRS broth and incubations were conducted statically at 37 °C for 24 h and 18 h, respectively. The culture at early stationary phase was harvested by centrifugation at 5000 rpm for 10 min at room temperature. The pellet was re-suspended in 0.85% NaCl (w/v) and the optical density at 600 nm (OD600) was determined to obtain a final concentration of 10^9^ CFU/ml^32^. The final culture solution that was kept at 4 °C until the administration was enumerated daily on MRS agar to confirm the dose administered to the mice which is10^8^ CFU/day.

### Animals and experimental procedure

**Figure 1:**
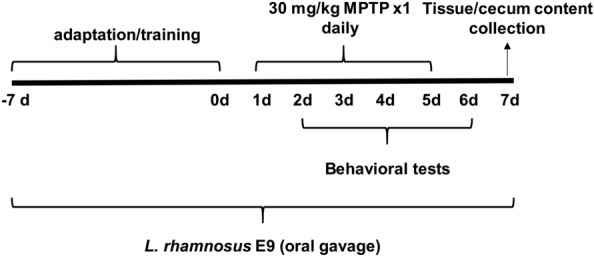
Schematic illustration of the experimental timeline.

All the procedures involving mice were reviewed and approved by the Local Ethics Committee for Animal Experiments with the protocol #311 (Kobay D.H.L. A.S., Ankara, Turkey) and all experiments were performed in accordance with relevant guidelines and regulations. Additionally the study conducted adheres to the ARRIVE guidelines^54^. A total of sixty healthy male C57BL/6 mice (8–10 week-old) were obtained from and housed as one in each cage at Kobay D.H.L. A.S. facility by following the guide for the care and the use of laboratory animals. Housing conditions were controlled at 22 ± 2 °C, 65–70% relative humidity with a 12 h light/dark cycle. Mice were fed the same water and mouse chow ad-libitum throughout the study. A week after acclimation to their surroundings in testing room, the animals were randomly divided into 4 groups; each group (n:15) was administered daily 100 μL of either 0.85% NaCl (Control and MPTP) or 10^8^ CFU/ml of *L. rhamnosus* E9 (E9) by oral gavage for fifteen days (Fig. 1). All mice except the Control groups were administered an intraperitoneal injection of 30 mg/kg MPTP-HCl (MedChemExpr, USA) daily for 5 consecutive days^55^. The Control groups were received equal volume of saline with the same procedure. All mice were sacrificed at the end of the experimental period, seven days after the first injection. After sacrifice, the cecum content was immediately placed on ice, and then kept at − 20 °C until processed for microbial DNA isolation. Animals in each group were randomly divided into three and the striatum of each animal was dissected out and rapidly snap-frozen for ELISA, preserved in 10% formaldehyde (Sigma-Aldrich, USA) for immunohistochemistry, or preserved in RNAlater (Ambion, USA) at 4 °C overnight and then at − 80 °C for RNA extraction.

### Behavioral procedures

After preadaptation and training, behavioral tests for motor functions were performed until the sacrifice. Tests were applied at the same time in the same order.

#### Open-field

For general locomotor activity and exploratory behavior of the mice, open field test was performed^23,56^. Each mouse was placed in the middle of a squared plexiglass box (40 × 40 × 40 cm) and allowed to freely move for 10 min while being monitored by an overhead camera. The records were analyzed to calculate the total distance traveled (mm) during the observation period and the average speed (mm/s) using an animal tracking software (ToxTrac)^57^.

#### Catalepsy bar test

The catalepsy of each mouse was evaluated^58^. The mice were positioned individually with forelimbs on a 4 cm-high horizontal bar and the maintenance time of the posture with fore/hindlimbs was recorded in seconds. The cut-off time was 180 s.

#### Wire hanging test

A wire-gripping test was performed to assess the whole body force^59^. Mice were suspended from a horizontal wire (30 cm high) by their forelimbs. Each mouse was scored from 1 to 4 based on the number of fore/hindlimbs used for gripping. Falling from the wire was scored as 0. The maximum time allowed was 60 s.

### Immunohistochemistry

After sacrification, brain and intestinal tissues were dissected intact and fixed in 10% neutral buffered formaldehyde. Sections were taken from the brain tissues in the coronal plane and from the intestinal tissues in the luminal plane. After tissue processing, paraffin embedding was performed and 4 micron sections were obtained for immunohistochemistry. Histopathological sections were kept in an oven at 70 °C for 30 min. and deparaffinized. The sections were then incubated in a graded alcohol series (100%, 100%, and 95%) for 5 min each and then rinsed under running tap water for 5 min. Antigen retrieval was performed in pH 6 citrate solution or pH 9 tris/EDTA solution depending on the antigen. The Leica Bond Max Autostainer (Shandon, Frankfurt, Germany) was used with the appropriate Leica brand secondary antibody kit. Primary antibodies against tyrosine hydroxylase (TH) (1/100, Biolegend, San Diego, CA, USA), SOX-10 (1/200, Biolegend, San Diego, CA, USA), GFAP (prediluted, Boster Lab, Pleasanton, CA, USA), Zonula Occluden-1 (ZO-1) (1/200, Boster Lab, Pleasanton, CA, USA), and Occludin (1/100, Booster Lab, Pleasanton, CA, USA) were stained using Leica Bond Max Autostainer (Frankfurt, Germany). The evaluation of immunohistochemistry was performed using different methods according to the tissue characteristics and the staining pattern. The detail of the evaluation for each antibody as follows; TH, from each brain tissue, 100 × microphotographs were obtained (Olympus, cellSens software, Tokyo, Japan) from the demonstrative striatum region and pixels in these photographs were converted into numerical values in the Image J software ("color threshold" tool) developed by the NIH (US Department of Health). SOX-10, the luminal sections were examined under a light microscope and positive nuclear reactions in submucosal and myenteric plexus were manually counted. GFAP, expression in the submucosal and the myenteric plexus localization representing the luminal neuronal axis was examined. While microphotographs were taken, mucosa was removed from the sections to rule out cross-reactions and to obtain a more definite result (to reduce the error of digital analysis). For the staining intensities on the wall outside the mucosa, pixel calculation was performed using the image J software. Occludin and ZO-1, evaluation of the epithelial tight junction proteins was performed on the mucosa. The staining intensity in the photographs were scored from 1 to 4 (score 1: barely visible staining, score 2: faint staining, score 3: neither strong nor faint staining, score 4: strong positive reaction).

### RNA isolation and gene expression analysis

Weighed brain and distal small intestine tissues were homogenized in ultra-pure-guanidine isothiocyanate (Sigma-Aldrich) using innuSPEED Lysis Tubes P in SpeedMill Plus (Analytik Jena, Germany). Total RNA was isolated and DNA contamination was removed using PureLink™ DNase Set (Invitrogen). Total RNA was converted into cDNA using High-Capacity cDNA Reverse Transcription Kit (Applied Biosystems) according to supplier’s protocol. qPCR was performed using the primers shown at Table S1. AMPIGENE qPCR Green Mix Hi-ROX (Enzo Life Sciences) was used under the following conditions: 95 °C for 2 min for initial denaturation, followed by 5 s (40 cycles) at 95 °C and 30 s at 57 °C. Data were generated in the final step at 95 °C for 15 s and melting curves (65 to 95 °C) were acquired at the end for each primer set. Relative gene expression was calculated by 2^−ΔΔCt^ method by normalizing gene expression to β-actin^60^.

### Enzyme-linked immunosorbent assay

Brain tissues for detection of dopamine and ROS level were homogenized in T-PER™ Tissue Protein Extraction Reagent and Halt Protease Inhibitor Cocktail, EDTA-Free (Thermo Scientific) using innuSPEED Lysis Tubes E in SpeedMill Plus (Analytik Jena, Germany)^61–63^. After centrifugation at max. speed for 5 min, the supernatant was used to carry out the experimental procedure as recommended by the manufacturer and the optical densities were measured at 450 nm.

### DNA extraction and gut microbiota analysis

After weighing, the cecum content was homogenized in PBS and total DNA was extracted using the QIAamp Fast DNA Stool-Mini Kit (Qiagen Sciences, MD) as described previously^32^. Briefly, a mechanical cell-disruption step was included by 0.1 mm glass-beads (Sigma-Aldrich) and specimens were beaten at maximum speed for six 1-min using the SpeedMill Plus (Analytik Jena, Germany) with intervals of 2-min on ice. Subsequently, a heat treatment step was carried out at 95 °C for 5 min and DNA was extracted. After DNA was quantified by NanoDrop spectrophotometer (Epoch, BioTek), 16S rRNA sequencing was performed using the Illumina NovaSeq 6000 at BMlabosis (Ankara, Turkey).

The V3-V4 region was amplified for library preparation, using specific primers (F-5ʹTCGTCGGCAGCGTCAGATGTGTATAAGAGACAGCCTACGGGNGGCWGCAG-3ʹ, R-5ʹGTCTCGTGGGCTCGGAGATGTGTATAAGAGACAGGACTACHVGGGTATCTAATCC-3ʹ). The Nextera XT index kit (Illumina, San Diego, CA) was used to attach unique identifiers to both 5′ and 3′ ends. The amplicons with equal quantities were pooled after PCR products were cleaned up using AMPure XP magnetic beads. Paired end reads sequenced on NovaSeq 6000 system underwent a quality-filtering and were trimmed with quality cut-off of Phred score of 20 by DADA2. OTUs were generated using DADA2 and data analysis was assessed in QIIME2 framework. The sequences were submitted to the Sequence Read Archive (SRA) database with the accession number of PRJNA1015441.

### Statistical analysis

The sample size was estimated to be five/group with 80% power (unpaired t-test; α = 0.05) to detect differences between the control and the treatment groups^21,64^. For microbiome, the statistical difference between groups was analyzed using the Mont Carlo test, a non-parametric test based on random permutations, in package ade4 generated by R 3.6.1 as described by de Carcer et al.^65–68^. The statistical difference of the between groups was evaluated with the randtest.between function in package ade4 of R 3.6.1. The zero (0) values were replaced with the limit of detection, which is assessed by the ratio of one to the lowest number of read in the data set. The Benjamini Hochberg procedure was applied to manage the false-discovery rate. The dominant genera that raised or diminished in abundance were identified by correspondence analysis in package ade4 of R 3.6.1^68^. Statistical difference for the rest of the analyses was performed with the Student’s* t* test or the Wilcoxon rank sum test using JMP Pro (SAS Institute Inc., Cary, NC) and introduced as mean ± SD. Statistical difference was ascertained at a P value of ≤ 0.05.

## Results and discussion

### *Lacticaseibacillus rhamnosus* E9 administration improved motor dysfunction in MPTP-induced mouse model of PD

**Figure 2:**
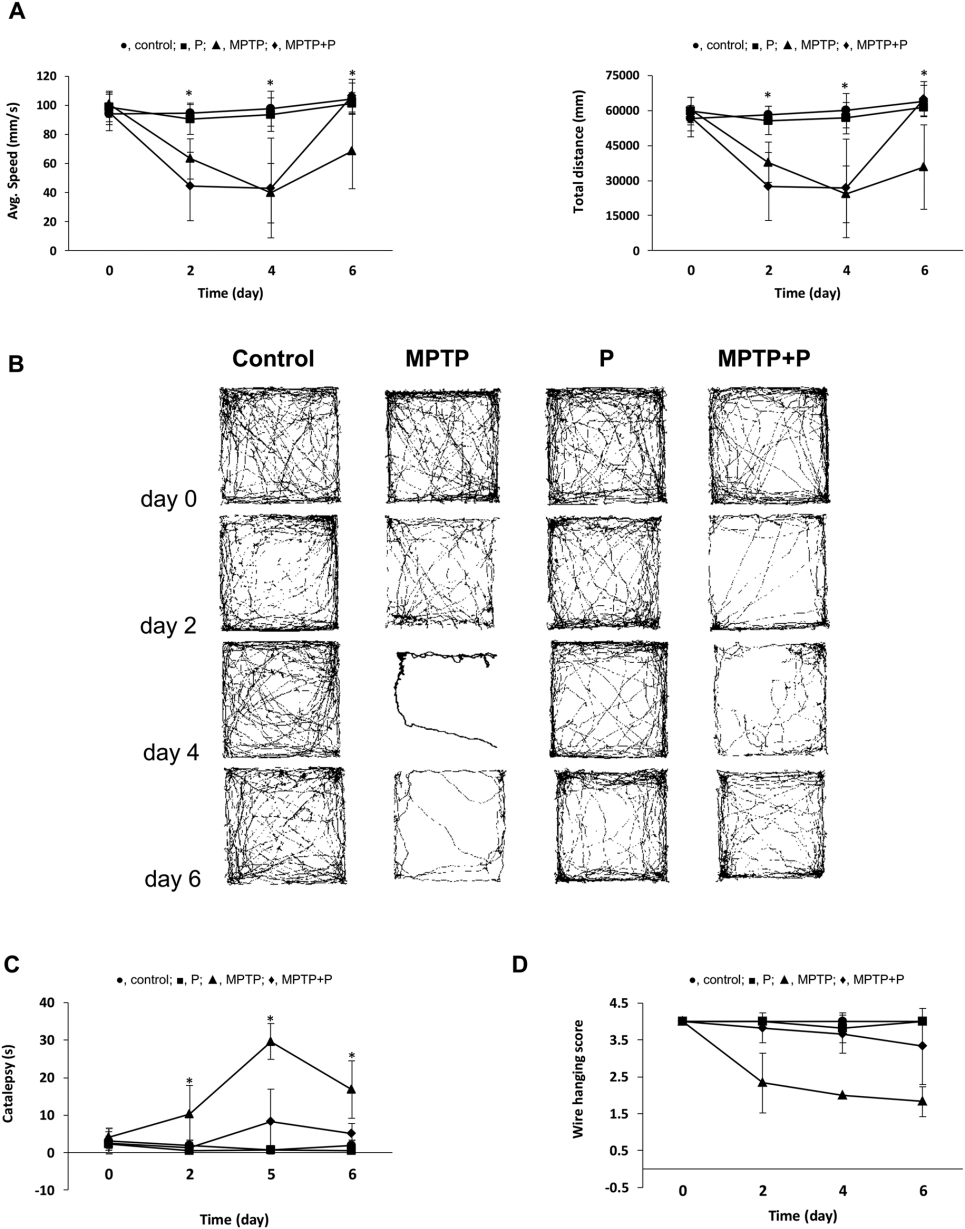
MPTP-induced behavioral alteration of mice in the open-field locomotion (**A**), catalepsy (**C**), and wire-hanging test (**D**). (**B**) Representative examples of moving pattern from each group of mice. MPTP mice were received (i.p.) 30 mg/kg MPTP-HCl daily for 5 consecutive days (MPTP and MPTP + P). Probiotic mice were administered 1 dose (10^8^ CFU/mouse/day) daily of *L. rhamnosus* E9 for fifteen days and sacrificed after the last dose (P and MPTP + P). filled circle, control; filled square, P; filled triangle, MPTP; filled diamond, MPTP + P. *p ≤ 0.05, control vs MPTP (n:6/group).

Motor dysfunction is one of the important characteristics of PD, which reflects dopamine depletion^7,8^. Behavioral tests were applied to measure the motor dysfunction that occurs due to dopaminergic damage in the mouse brain after MPTP injection. The general locomotor activity and exploratory behavior of mice were tested by open field test. A standard bar test and a wire grip test were administered to evaluate catalepsy and whole body strength, respectively. The effect of MPTP was reflected in the behavioral tests one day after MPTP injection and the normal locomotor activity, the exploratory behavior, and the posture maintenance ability of the mice were impaired. These behavioral findings are consistent with the motor symptoms in MPTP-induced PD mouse models as part of their response to MPTP administration^69–71^. MPTP injection significantly decreased the locomotor activity and animals' exploratory behavior in both the MPTP and the probiotic groups. It can be seen in the open-field test with reduced average speed and overall traveled distance in 10 min (Fig. 2). However, E9 administration helped to recover the locomotor activities faster than the MPTP mice that were not received probiotics. Catalepsy and wire hanging test results (Fig. 2) showed that the total time to correct an imposed posture resulting from muscular rigidity and muscle strength were not affected in the E9 group as much as in the MPTP group (*p* < 0.05). These results show that E9 administration effectively alleviates the impaired motor functions in MPTP-induced mouse model of PD. In a study applying another strain of the same species as we used in this study (*L. rhamnosus* HA-114), probiotic treatment had no significant effect on behavioral deficits in an OHDA-induced PD mouse model, showing us the strain specific impact of probiotics^72^. On the other hand, there are studies stated that brain damage may not be reflected in behavioral tests to the same extent or that this reflection may vary depending on the behavioral test applied^55,73–75^.

### *Lacticaseibacillus rhamnosus* E9 administration attenuated dopaminergic neuronal death in MPTP-induced mouse model of PD

**Figure 3:**
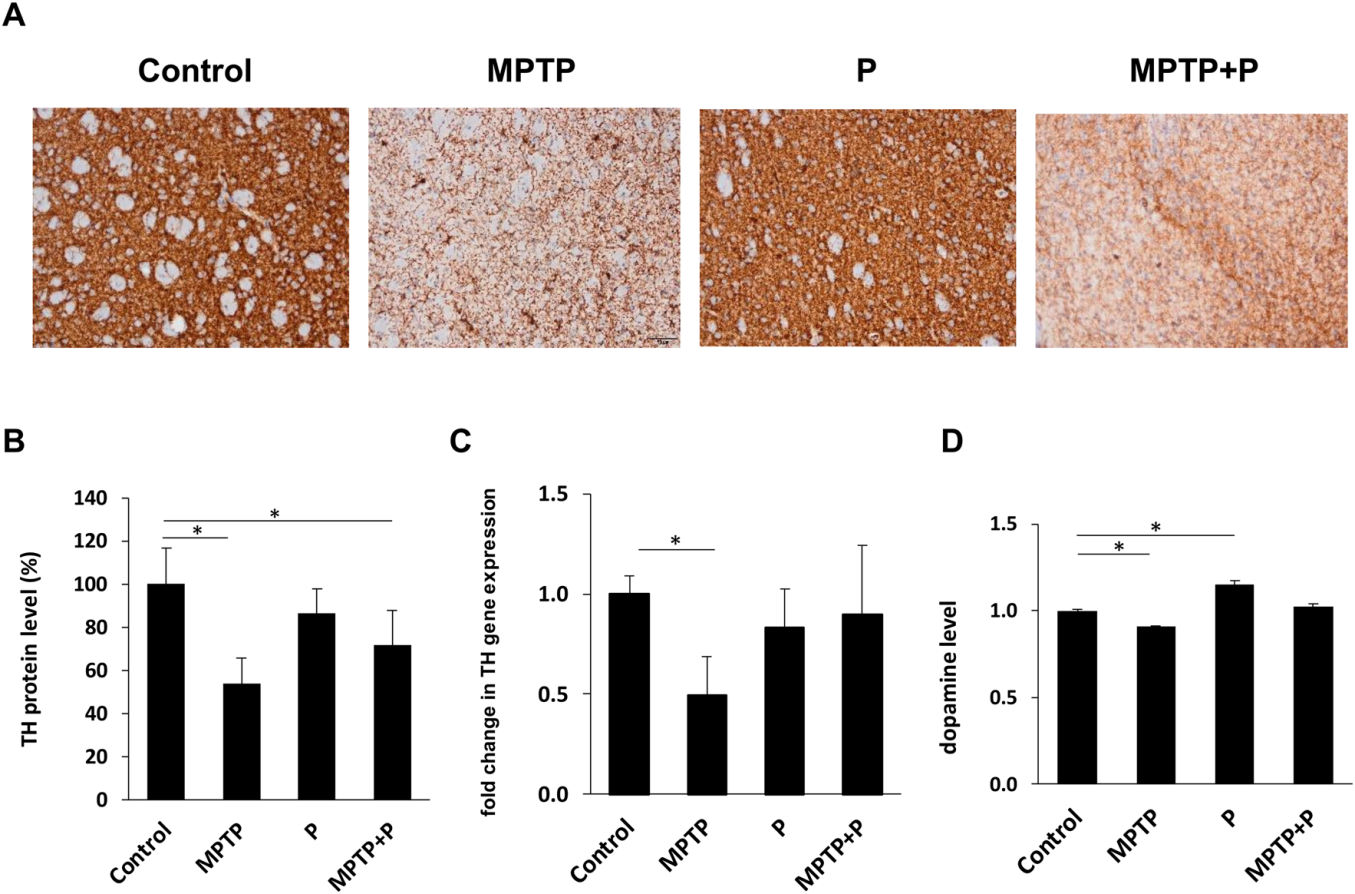
Impact of *Lacticaseibacillus rhamnosus* E9 administration on striatal Tyrosine hydroxylase (TH) protein (**A**, **B**) and gene (**C**) expression level and dopamine level (**D**) in MPTP-induced mouse model of PD compared to the control. MPTP mice were received (i.p.) 30 mg/kg MPTP-HCl daily for 5 consecutive days (MPTP and MPTP + P). Probiotic mice were administered 1 dose (10^8^ CFU/mouse/day) daily of *L. rhamnosus* E9 for fifteen days and sacrificed after the last dose (P and MPTP + P). *p ≤ 0.05, control vs MPTP (n:4–5/group).

Dopaminergic neural damage occurring in PD patients leads to a decrease in dopamine levels, and therefore, in the levels of dopamine receptors, dopamine transporters, and the tyrosine hydroxylase enzyme (TH) that synthesizes dopamine^76^. Decrease in dopamine level is one of the pathological changes occurring in PD and TH, the rate-limiting enzyme of dopamine synthesis, plays a critical role in this pathology^7,8^. TH, hence, has been recognized as a biomarker of dopaminergic neurons and measured to validate the success of the PD animal models generated to examine PD pathology and to develop possible therapeutics^77,78^. After striatum was dissected from the brain tissues, levels of TH and dopamine were measured to evaluate the dopaminergic neuronal death in mice. (Fig. 3). A significant decrease in TH expression was detected in striatum of the MPTP mice, indicating an impaired dopamine synthesis by MPTP injection. Immunohistochemistry showed that TH expression that was decreased significantly (*p* < 0.05) after MPTP application (54 ± 11%) was increased by E9 administration (72 ± 16%). Similar results were seen in striatal TH gene expression as well. Additionally, dopamine level in the striatal tissues was decreased significantly (*p* < 0.05) after MPTP application in parallel with the level of TH enzyme, a dopamine producer. Dopamine in the mice administered E9 was not significantly different than the control mice. The results demonstrated that E9 has a neuroprotective effect on MPTP-induced mouse model of PD by preventing dopamine loss.

**Figure 4:**
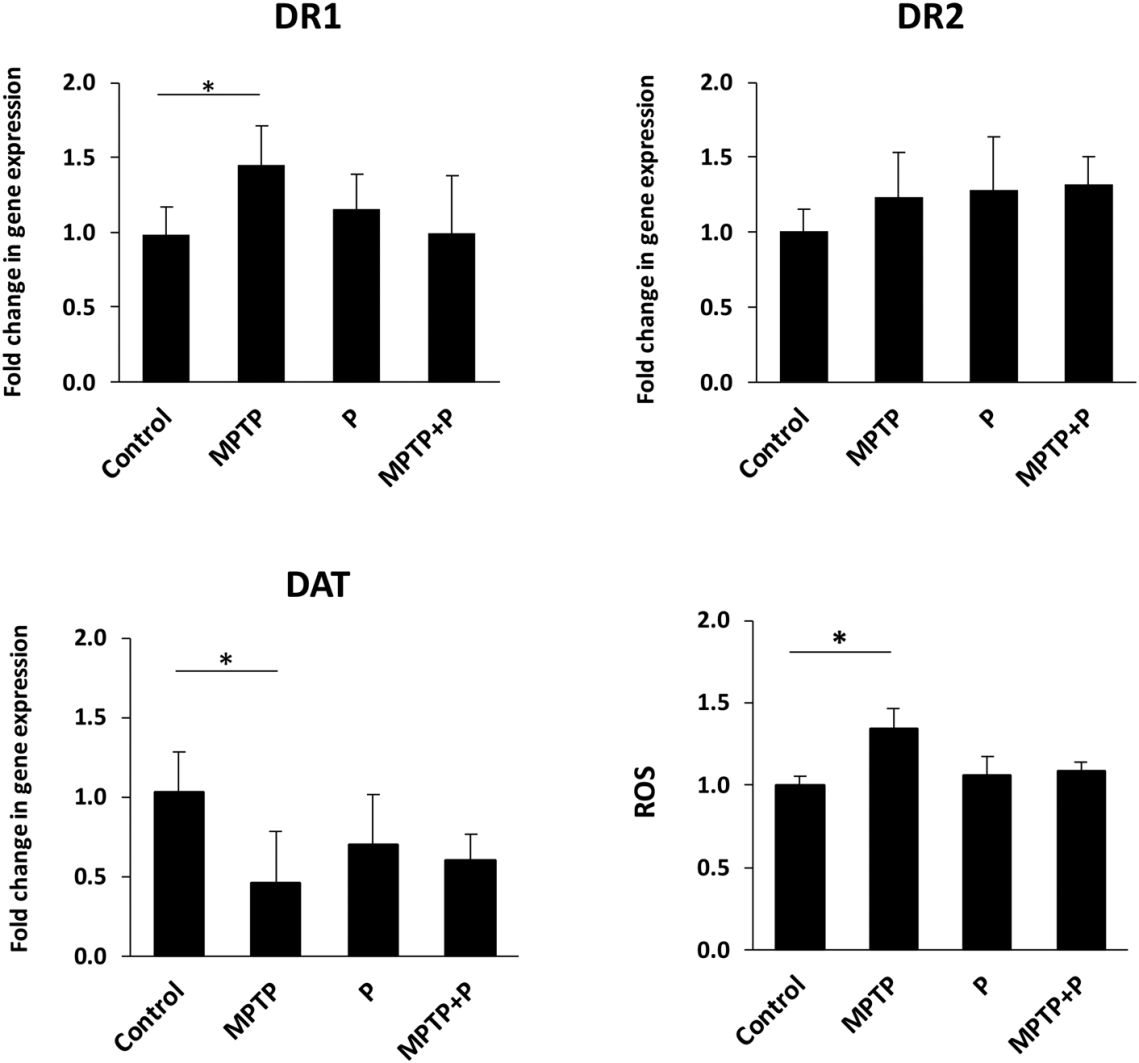
Impact of *Lacticaseibacillus rhamnosus* E9 administration on striatal gene expression of Dopamine Receptor 1 (*DR1*), Dopamine Receptor 2 (*DR2*), and Dopamine Transporter (*DAT*) and Reactive Oxygen Species (ROS) in MPTP-induced mouse model of PD compared to the control. MPTP mice were received (i.p.) 30 mg/kg MPTP-HCl daily for 5 consecutive days (MPTP and MPTP + P). Probiotic mice were administered 1 dose (10^8^ CFU/mouse/day) daily of *L. rhamnosus* E9 for fifteen days and sacrificed after the last dose (P and MPTP + P). *p ≤ 0.05, control vs MPTP (n:4–5/group).

Dopamine receptors (DRs) comprise D1-like receptors, which include DR1 and DR5, and D2-like receptors, which include DR2, DR3, and DR5^79^. Striatal dopamine receptors mostly consist of DR1 and DR2. Dopamine transporter (DAT) protein is in charge of dopamine uptake and valuable in the clinical diagnosis of PD^76^. MPTP injection has been shown to reduce DAT levels in brain tissue of PD animals as part of the pathophysiology of PD^80^. In this study, the expression level of *DR1*, *DR2*, and *DAT* genes of the striatal tissues were examined. While the expression of *DR1* significantly (*p* < 0.05) increased in the MPTP-induced mice, *DR2* showed no statistical differences from the control in all samples evaluated (data not shown). Reports on modification in the level of dopamine receptors has been controversial in both PD animal models and PD patients^76,79,81–84^. A decrease, an increase, or no change have been documented in MPTP-induced animal models of PD. The *DAT* expression in MPTP-induced mice was reported for the first time in this study and found to be significantly (*p* < 0.05) decreased in parallel with the loss of dopaminergic neurons in MPTP mice (Fig. 4). The expression levels of *DR1* and *DAT* in mice administered E9 showed no significant differences compared to the control.

### *Lacticaseibacillus rhamnosus* E9 administration reduced reactive oxygen species (ROS) formed in striatal tissue

Factors affecting the neural degeneration in sporadic PD patients include an increase in the production of free radicals and oxidative stress^7,8^. Reactive oxygen and nitrogen are mainly produced in mitochondria as a result of aerobic metabolism. Incomplete induction of oxygen and nitrogen causes the formation of radicals such as superoxide radical (O2-), hydrogen peroxide (H2O2), and nitric oxide (NO). These radicals can cause oxidative damage to proteins, membranes, and DNA^85^. Oxidative stress has found to be high in the brain tissues of PD patients^86^. Reactive oxygen species (ROS) in the brain was determined to evaluate oxidative damage resulting from inflammation. In the MPTP group, a significant (*p* < 0.05) increase was observed in the ROS level compared to the control group. It was not significantly different in mice administered E9 from the control. E9 reduced the level of ROS formed in the striatal tissue by MPTP injection (Fig. 4). ROS has been shown to correlate positively with the inflammation in the brain of PD mice^87^. Additionally, studies suggest that production of ROS leads to misfolding of α-syn in the brain and contributes to development of synucleinopathies including PD^88^. Lactobacilli administration has been previously shown to prevent α-syn aggregation in substantia nigra of MPTP-induced mice by inhibiting the oxidative damage and the inflammation in brain^87^. Further investigation of the impact of E9 on inflammation in MPTP-induced mouse model of PD would provide more insight into the mechanism of action by E9 on oxidative stress and neuroprotection.

### *Lacticaseibacillus rhamnosus* E9 administration enhanced intestinal barrier integrity in MPTP-induced mouse model of PD

**Figure 5:**
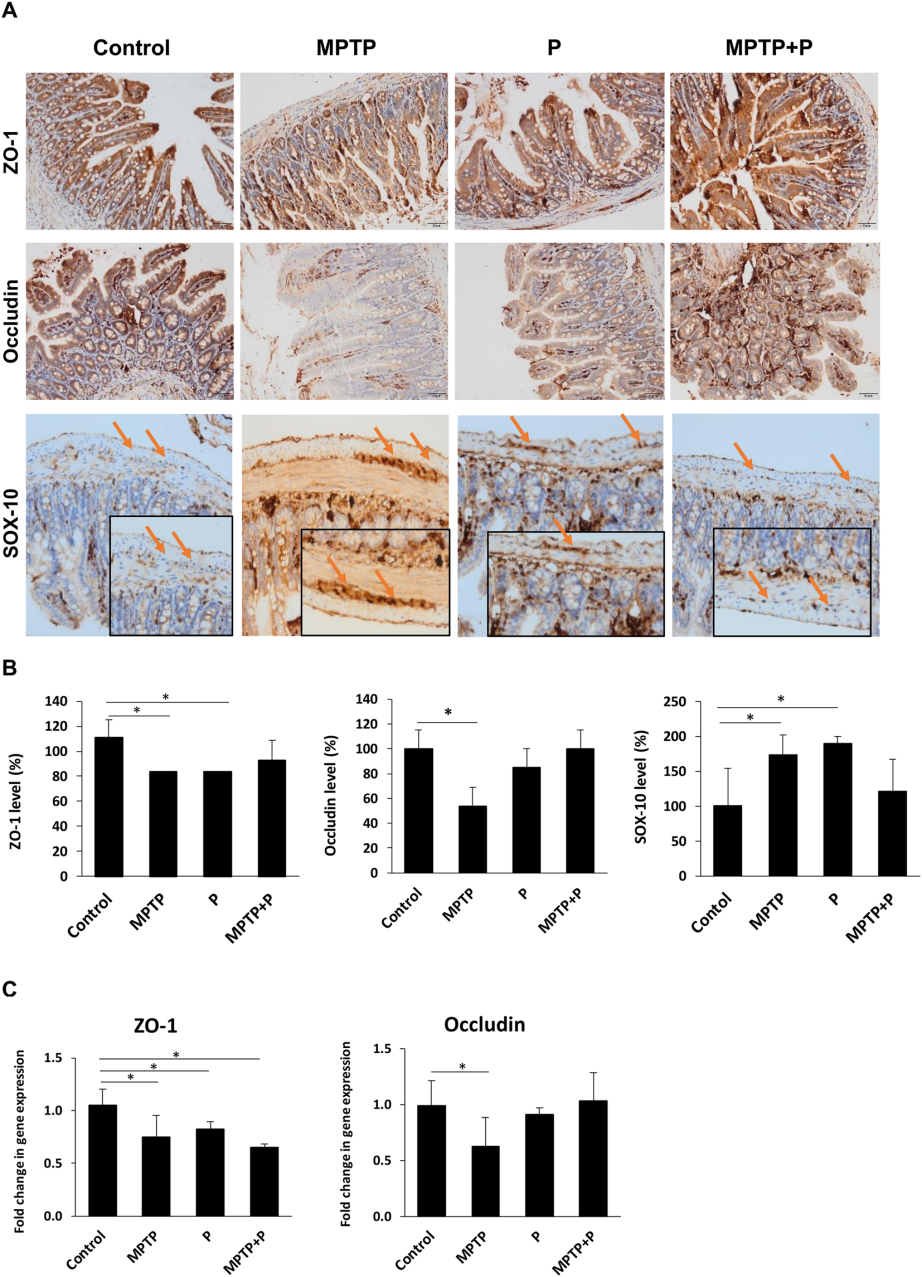
Effect of *Lacticaseibacillus rhamnosus* E9 administration on tight junction proteins and SOX-10 in ileum of MPTP-induced mouse model of PD compared to the control. (**A**) Immunohistochemical staining of ileal ZO-1 (100 ×), Occludin (100 ×) (staining intensity is evaluated in ideal mucosa), and SOX-10 (200 ×, inset-4000 ×) (Arrows are nuclear positivity in myenteric plexus). (**B**) Protein expression of ileal ZO-1, Occludin, and SOX-10 in PD and control mice. (**C**) Gene expression of ileal ZO-1 and Occludin in PD and control mice. MPTP mice were received (i.p.) 30 mg/kg MPTP-HCl daily for 5 consecutive days (MPTP and MPTP + P). Probiotic mice were administered 1 dose (10^8^ CFU/mouse/day) daily of *L. rhamnosus* E9 for fifteen days and sacrificed after the last dose (P and MPTP + P). *p ≤ 0.05, control vs MPTP (n:4–5/group).

One of the gastrointestinal symptom commonly seen in PD patients is leaky gut syndrome due to increased intestinal permeability^2,89^. Pro-inflammatory cytokines and glial markers have been reported to increase in colonic biopsies collected from PD patients and that these levels correlate with the duration of the disease^90^. In addition to the intestinal inflammation, a significant increase in the intestinal permeability has been observed in PD patients compared to their controls^18^. A weakened intestinal barrier causes translocation of bacteria and bacterial components which leads to inflammation and oxidative stress in the enteric nervous system^91^. To assess changes in the impact on tight junction proteins (TJPs), that provide intercellular connection and strengthen the intestinal epithelium of the E9 probiotic strain, we examined the ileal expression of ZO-1 and Occludin proteins in MPTP mice after probiotic administration. We observed a significant (*p* < 0.05) decrease in expression of both ZO-1 and Occludin protein and gene in MPTP mice (Fig. 5). Occludin gene and protein expression in probiotic MPTP mice was detected at the same level as the control. E9 administration improved the intestinal integrity. The expression of TJPs was not different than the control. Enteric glial markers, GFAP and SOX-10, were also evaluated. Expression of SOX-10 was significantly (*p* < 0.05) elevated in MPTP mice while it was not different than control in E9 administered mice (Fig. 5). Although GFAP showed no statistical differences from the control in all samples evaluated (data not shown), SOX-10/GFAP ratio, which induces intestinal motor dysfunctions and contributes to enteric inflammatory responses, was increased from 0.9 to 2.8 in MPTP mice but it was 1.5 in E9 administered mice^92^. An upregulation in SOX-10 expression has been shown also in colon biopsies collected from PD patients^90^. While the increase in GFAP and SOX-10 levels have found to be in parallel with the level of inflammation, not enough data has been reported about the GFAP level in PD patients^92^. In another study, rotenone induced mice were received *L. plantarum* CCFM405 for 9 weeks and the gene expression of TJPs in colon was examined^70^. *L. plantarum* administration ameliorated the decrease in gene expression of *ZO*-1 and *Occludin* after rotenone application. It was emphasized that probiotics impact the brain-gut axis by rearranging the gut microbiota and that probiotic administration can be an alternative strategy in PD management. The decrease in the level of ZO-1 in the probiotic only group of mice could be related to the increased intestinal SOX-10 level which is associated with inflammation^92^. Pattern recognition receptors such as Toll-like receptors (TLRs) contribute to the interaction between intestinal cells, gut microbiota, and the immune system^93^. They are involved in maintaining the tight junctions between intestinal epithelial cells^94^. The expression of TLRs in the intestine may differ depending on the probiotic strain due to both their different surface structure and their interaction with the commensals^95–99^. Accordingly, intestinal inflammation could be indirectly affected^100–102^.

### *Lacticaseibacillus rhamnosus* E9 administration remodulated the cecal microbiota altered in MPTP-induced mouse model of PD

Chronic inflammatory state, an imbalance between pro-inflammatories and anti-inflammatories has been thought to be the main cause of PD, which accelerates aging and damages neural cells^85,103^. Gastrointestinal microbiota is one of the main factors affecting and even regulating the immune system in the host^104,105^. Gut microbiota is also known to communicate with the brain and this communication maintains the gastrointestinal and the nervous system homeostasis along with the immune system^13–15^. A disruption in the communication within the gut-brain axis is associated with various neurodegenerative diseases including PD^16^. As a matter of fact, clinical studies have revealed dysbiosis in the intestinal microbiota of PD patients^19–21^. Recent research shows that histological and behavioral findings in PD are associated with dysbiosis in the gut microbiome and the potential of probiotics to prevent PD progression is one of the latest innovations in the field of neurodegenerative diseases^70,106,107^. However, studies unrevealing the mechanism of probiotic action on PD pathogenesis with the association of the brain-gut axis is limited. In our study, we demonstrated the changes in the microbial composition in MPTP-induced mouse model of PD and impact of E9 administration on the altered cecal microbiota by MPTP injection using 16S rRNA sequencing. As a result of sequencing, a total of 1,776,677 filtered reads were obtained from the cecal content samples of 20 mice. The number of reads per sample ranged from 47,182–168,617 with an average number of 88,833.85 reads/sample. After assigning the each read to a taxonomic level, 18 phyla, 26 classes, 54 orders, 108 families and 257 genera were identified. To assess whether sufficient sequence reads had been obtained to accurately determine the diversity of organisms present, Chao1 and Shannon index were computed (Fig. S1). Additionally, a principal coordinate analysis (PCoA) plots using weighted Unifrac distances for beta diversity were generated.

**Figure 6:**
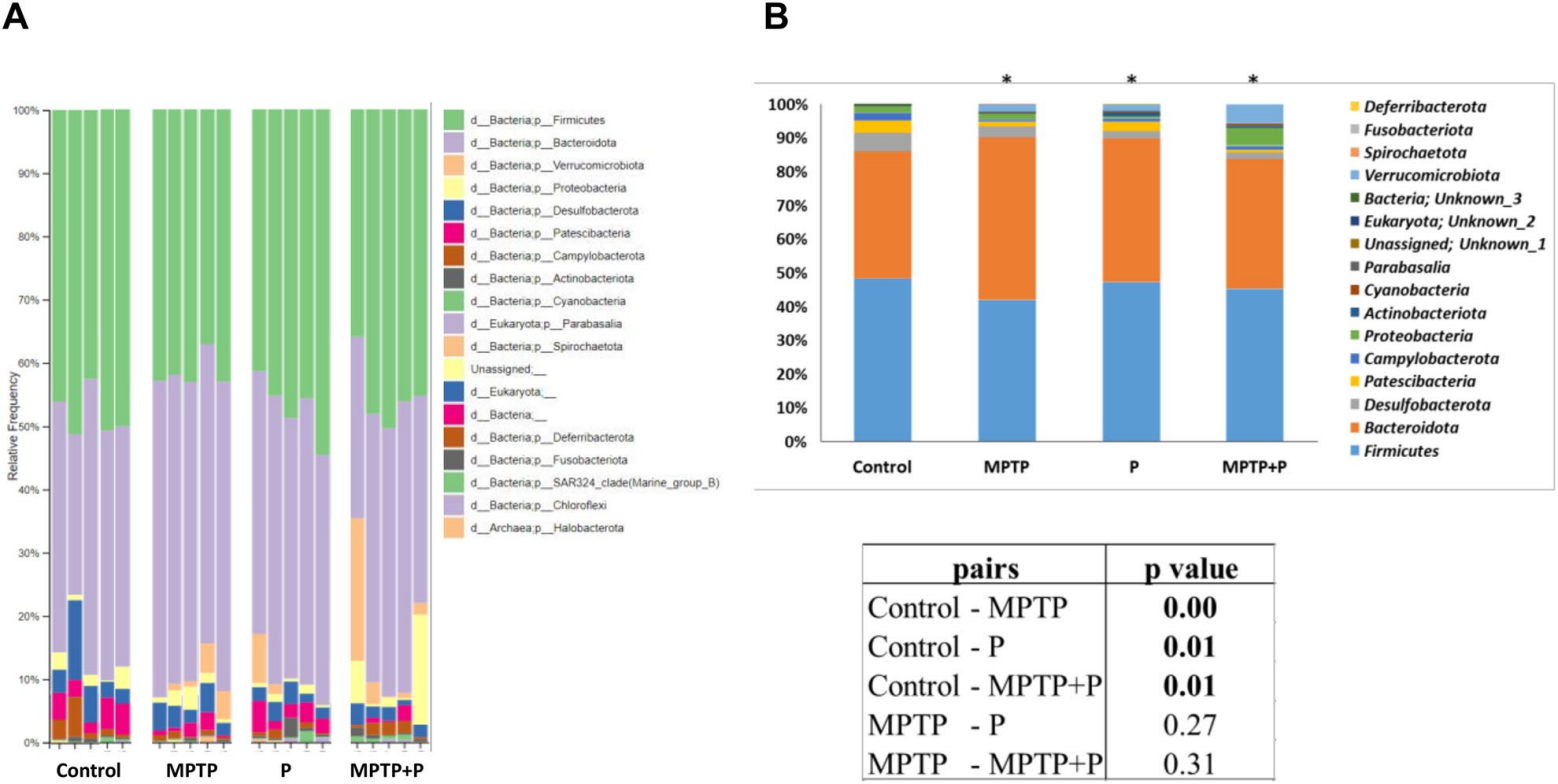
Comparison of predominant phyla in the microbiota of mice cecums. (**A**) Change of individual mouse cecum microbiota at the phylum level after MPTP and/or probiotic application. (**B**) Predominant phylum in the cecum microbiome of mice and pair wise comparison of each group. Statistical p-values were assessed using a Monte-Carlo test with 10,000 replicates. MPTP mice were received (i.p.) 30 mg/kg MPTP-HCl daily for 5 consecutive days (MPTP and MPTP + P). Probiotic mice were administered 1 dose (10^8^ CFU/mouse/day) daily of *L. rhamnosus* E9 for fifteen days and sacrificed after the last dose (P and MPTP + P). *p ≤ 0.05, (n:5/group).

The cecum microbiota of the MPTP mice fed with/without E9 was evaluated at the 3.5 h after the last administration, the time lactobacilli bolus reaches the cecum, and compared with the healthy control^32^. A Monte-Carlo test with 10,000 replicates was utilized to identify the groups that were significantly (*p* < 0.05) different from each other. The overall microbial composition at the phylum level differed significantly (*p* < 0.05) from the control in the MPTP injected mice (Fig. 6). The dominant phyla in rank order of the cecum microbiota were *Firmicutes* and *Bacteroidota*, which constitute 84–90% of the total microbiome (Fig. 6 and Table S2). While the dominant phylum in healthy and E9 mice was *Firmicutes* with 48% and 45%, respectively, the abundance of *Firmicutes* decreased significantly (*p* < 0.05) to 41% in the MPTP mice. The dominant phylum was *Bacteroidota* with 48% in the MPTP mice. The predominance of *Bacteroidota* in control and E9 mice was determined as 37.9% and 38.5%, respectively and was not significantly different from each other. *Firmicutes* and *Bacteroidota* are the two major phyla detected in human and rodent gastrointestinal microbiome, and the ratio of these two phyla has been found to be relevant to human health^108^. The *Firmicutes*/*Bacteroidota* ratio has been shown to be reduced in patients with Crohn's disease, ulcerative colitis, and infectious colitis, where the intestinal epithelial barrier is damaged and general inflammation is stimulated^109^. In our study, while the *Firmicutes*/*Bacteroidota* ratio was 1.3 and 1.2 in control and E9 mice, respectively, it decreased to 0.9 in the MPTP mice, which was statistically (*p* < 0.05) different from the healthy control and E9 mice. E9 administration increased the *Firmicutes*/*Bacteroidota* ratio. In a study conducted with fecal samples obtained from PD patients, the microbial composition was compared with the healthy controls and a decrease in the abundance of *Firmicutes* but an increase in the *Bacteroidata* abundance was reported^110^.

**Figure 7:**
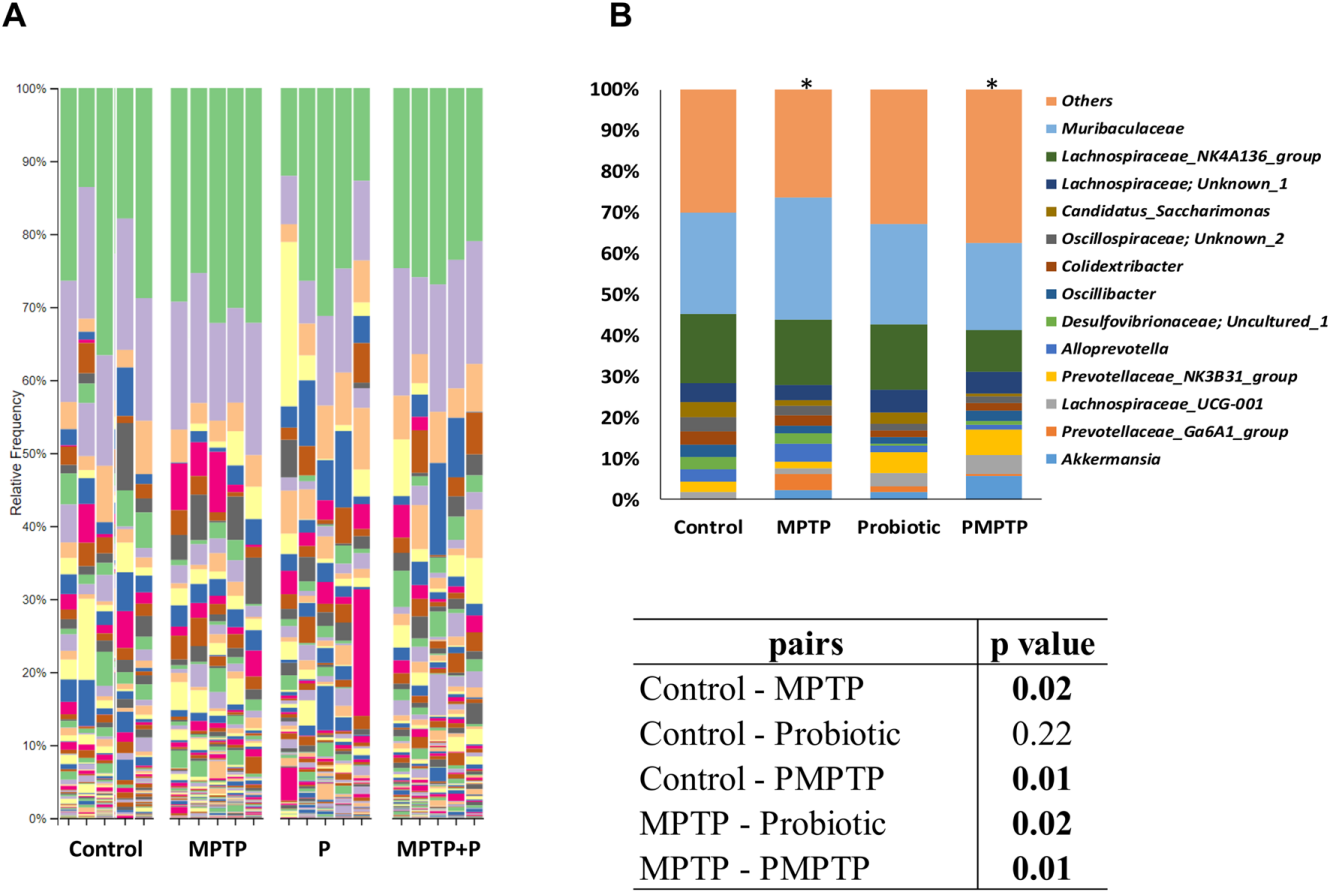
Comparison of predominant genera in the microbiota of mice cecums. (**A**) Change of individual mouse cecum microbiota at the genus level after MPTP and/or probiotic application. (**B**) Predominant genera in the cecum microbiome of mice and pair wise comparison of each group. Statistical p-values were assessed using a Monte-Carlo test with 10,000 replicates. MPTP mice were received (i.p.) 30 mg/kg MPTP-HCl daily for 5 consecutive days (MPTP and MPTP + P). Probiotic mice were administered 1 dose (10^8^ CFU/mouse/day) daily of *L. rhamnosus* E9 for fifteen days and sacrificed after the last dose (P and MPTP + P). *p ≤ 0.05, (n:5/group).

**Figure 8:**
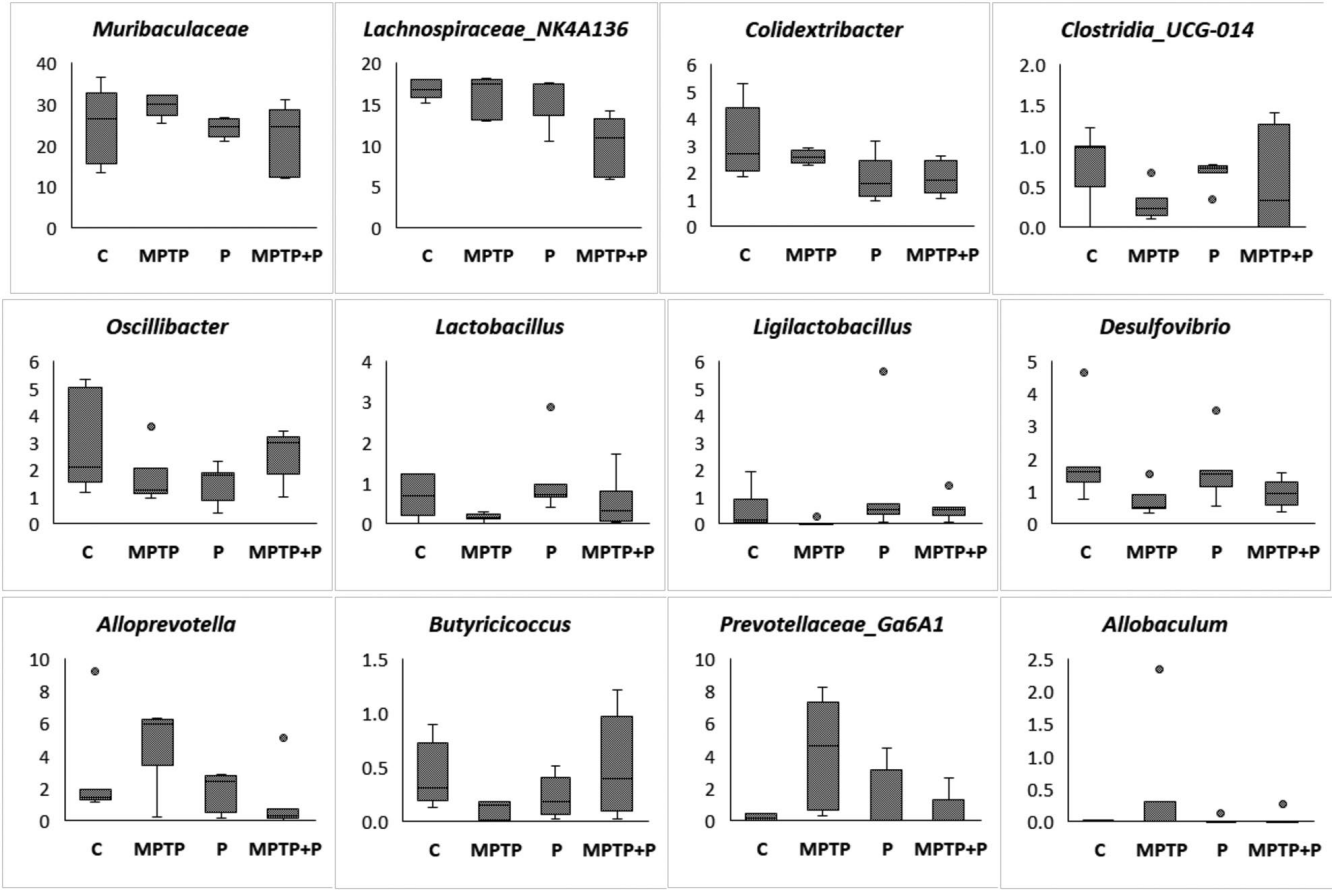
The abundance of genera (%) changed in mouse cecum microbiota after MPTP and/or probiotic application. MPTP mice were received (i.p.) 30 mg/kg MPTP-HCl daily for 5 consecutive days (MPTP and MPTP + P). Probiotic mice were administered 1 dose (10^8^ CFU/mouse/day) daily of *L. rhamnosus* E9 for fifteen days and sacrificed after the last dose (P and MPTP + P). (n:5/group).

The overall microbial composition at the genus level in MPTP injected groups differed significantly (*p* < 0.05) from each other (Figs. 7, 8, and Fig. S2). The dominant genera of the cecum microbiota at the genus level were *Muribaculaceae*, previously known as S24-7, and *Lachnospiraceae*_NK4A136_group, and these genera together constitute 31–46% of the total microbiota (Table S3)^111^. While the abundance of *Muribaculaceae* increased from 24.6 to 29.8% in MPTP mice, it decreased to 21.4% in mice fed with E9. *Muribaculaceae*, for which there is not much information in the literature, is known as mucus-derived monosaccharide users in the gut^111^. Similarly, microbiome analysis of fecal samples from MPTP-induced PD mice demonstrated the *Muribaculaceae* population to be increased^112^. The predominance of *Lachnospiraceae*_NK4A136_group was between 16.0% and 16.9% in control and MPTP mice; however, it decreased to 10% in the mice administered E9. In a study investigating the relationship between the motor dysfunctions in PD and the gut microbiota, *Lachnospiraceae*_NK4A136 abundance was found to be increased in patients experiencing motor complications during chronic dopamine replacement therapy^113^. The predominance of *Colidextribacter*, which decreased in E9-administered mice, was reported to be associated with aging and showed a significant increase in the murine microbiota during aging^114^. Additionally, they were positively correlated with cognitive fragility. The abundance of *Clostridia*_UCG-014, which was negatively correlated with the cognitive fragility in studies exploring the gut-brain axis, was decreased in MPTP mice in our study^114^. E9 administration restored back to the same level as the control. The level of *Oscillibacter*, which is also reported to have a negative correlation with cognitive performance and inflammation, decreased in MPTP mice, while it was restored back to the same level as the control in mice received E9^115^. Likewise, the predominance of *Lactobacillus* and *Ligilactobacillus*, previously belonged to *Lactobacillus,* were decreased in MPTP mice but increased in mice fed with E9 to the same level as the control^116^. These genera consist of strains commonly used as probiotics and various health benefits have been associated with their use as probiotics^95,117–119^. *Desulfovibrio*, negatively correlated with irritable bowel syndrome, was found to be associated with PD^120^. In our study, it positively correlated with the level of TH enzyme responsible for dopamine synthesis. The increase in the abundance of *Alloprevotella*, reported to be positively correlated with dyskinesia, was decreased by E9 administration^121^. *Butyricicoccus* is a producer of butyrate, a short-chain fatty acid used by enterocytes as an energy source and suppressing inflammation as well as enhancing the intestinal barrier^122,123^. PD-derived fecal samples were found to contain less butyrate producing bacteria^20,110^. A study with clinical phenotyping approach including metabolomics and metagenomics in PD patients reported that low level of butyrate was associated with the worse postural instability gait-disorder scores in PD^123^. The *Butyricicoccus* abundance decreased in MPTP mice while increased in mice fed with E9. The predominance *of Prevotellaceae* Ga6a1 group, associated with anxiety like behavior in rats, was increased by MPTP injection but was decreased in E9 mice^124^. Based on Spearman’s ρ correlation analysis TH level in striatum was positively correlated with *Desulfovibrio* and *Clostridia*_UCG-014 abundance, but negatively correlated with *Allobaculum* (*p* < 0.0001). Additionally, ileal SOX-10 level showed a negative correlation with *Oscillibacter* and *Helicobacter* abundance (*p* < 0.0001). These results suggest that the composition of the cecal microbiota, which was altered by MPTP injection and associated with dopamine synthesis, was likely modified and restored by E9 administration.

E9 strain used in this study is known to be a high EPS producer^43–45^. EPSs are shown to have immunomodulatory effect and regulate the gut microbiota and the dopaminergic system in mice^46–50^. Modulating the gut microbiota, taking role in the neurodegenerative diseases, by E9 could be associated with the ability of E9 to produce high amount of EPS known to have biological activities such as anti-proliferative and anti-inflammatory activity, and neuroprotective effect^43–45,51^ Since the impact of EPSs on PD has not been studied yet, further investigation on E9 EPSs for their impact on immunomodulation and PD development could be significant to understand the mechanism of probiotic action.

## Conclusion

In conclusion, we provide a comprehensive study investigating the effect of probiotics on PD with the association of the gut-brain axis. Overall our results indicated that *L. rhamnosus* E9 administration effectively exerted a neuroprotective effect on dopaminergic neural loss in MPTP-induced mouse model of PD. E9 improved the behavioral dysfunction of PD mice, inhibited MPTP-induced dopaminergic neural damage, oxidative stress, and gut barrier impairment and the neuroprotective mechanism of action involved the rearranging the gut microbiota. E9, an EPS producer lactobacilli strain, is a promising oral supplement as an alternative strategy for PD management via impacting the gut-brain axis. The effect of E9 on gut-brain axis-related inflammation also need to be investigated in future studies to reveal the full mechanism of action.

### Supplementary Information

Supplementary Information.

## References

[1] Lindqvist D (2012). Non-motor symptoms in patients with Parkinson’s disease: Correlations with inflammatory cytokines in serum. PLoS ONE.

[2] Anderson G, Carvalho A (2016). Gut permeability and microbiota in Parkinson’s disease: Role of depression, tryptophan catabolites, oxidative and nitrosative stress and melatonergic pathways. Curr. Pharm. Des..

[3] Dorsey ER (2007). Projected number of people with Parkinson disease in the most populous nations, 2005 through 2030. Neurology.

[4] Foundation, P. Understanding Parkinson’s. *Parkinson’s Foundation Parkinson’s Prevalence Project* (2021). https://www.parkinson.org/Understanding-Parkinsons/Statistics.

[5] Medicine, J. H. Young-Onset Parkinson’s Disease. *Medicine, Johns Hopkins* (2021). https://www.hopkinsmedicine.org/health/conditions-and-diseases/parkinsons-disease/youngonset-parkinsons-disease.

[6] Dorsey ER, Bloem BR (2018). The Parkinson pandemic: A call to action. JAMA Neurol..

[7] Taylor JM, Main BS, Crack PJ (2013). Neuroinflammation and oxidative stress: Co-conspirators in the pathology of Parkinson’s disease. Neurochem. Int..

[8] Li Y, Liu WZ, Li L, Hölscher C (2017). D-Ala2-GIP-glu-PAL is neuroprotective in a chronic Parkinson’s disease mouse model and increases BNDF expression while reducing neuroinflammation and lipid peroxidation. Eur. J. Pharmacol..

[9] Houser MC, Chang J, Factor SA, Molho ES, Zabetian CP (2018). Stool immune profiles evince gastrointestinal inflammation in Parkinson’s disease. Mov. Disord..

[10] Hopfner F (2017). Gut microbiota in Parkinson disease in a northern German cohort. Brain Res..

[11] Schapira AHV, Chaudhuri KR, Jenner P (2017). Non-motor features of Parkinson disease. Nat. Rev. Neurosci..

[12] Lima IS (2023). Gut dysbiosis: A target for protective interventions against Parkinson’s disease. Microorganisms.

[13] Pellegrini C, Antonioli L, Colucci R, Blandizzi C, Fornai M (2018). Interplay among gut microbiota, intestinal mucosal barrier and enteric neuro-immune system: A common path to neurodegenerative diseases?. Acta Neuropathol..

[14] Santos SF, De Oliveira HL, Yamada ES, Neves BC, Pereira A (2019). The gut and Parkinson’s disease: A bidirectional pathway. Front. Neurol..

[15] Rani L, Mondal AC (2021). Unravelling the role of gut microbiota in Parkinson’s disease progression: Pathogenic and therapeutic implications. Neurosci. Res..

[16] Morais LH, Schreiber HL, Mazmanian SK (2021). The gut microbiota–brain axis in behaviour and brain disorders. Nat. Rev. Microbiol..

[17] Hasegawa S (2015). Intestinal dysbiosis and lowered serum lipopolysaccharide-binding protein in Parkinson’s disease. PLoS ONE.

[18] Schwiertz A (2018). Fecal markers of intestinal inflammation and intestinal permeability are elevated in Parkinson’s disease. Park. Relat. Disord..

[19] Hill-Burns EM (2017). Parkinson’s disease and Parkinson’s disease medications have distinct signatures of the gut microbiome. Mov. Disord..

[20] Unger MM (2016). Short chain fatty acids and gut microbiota differ between patients with Parkinson’s disease and age-matched controls. Park. Relat. Disord..

[21] Sun MF (2018). Neuroprotective effects of fecal microbiota transplantation on MPTP-induced Parkinson’s disease mice: Gut microbiota, glial reaction and TLR4/TNF-α signaling pathway. Brain. Behav. Immun..

[22] Sampson TR (2016). Gut microbiota regulate motor deficits and neuroinflammation in a model of Parkinson’s disease. Cell.

[23] Biju KC (2018). Methylene blue ameliorates olfactory dysfunction and motor deficits in a chronic MPTP/probenecid mouse model of Parkinson’s disease. Neuroscience.

[24] Armstrong MJ, Okun MS (2020). Diagnosis and treatment of Parkinson disease: A review. JAMA J. Am. Med. Assoc..

[25] Yang X (2023). Effect of *Lacticaseibacillus paracasei* strain Shirota supplementation on clinical responses and gut microbiome in Parkinson’s disease. Food Funct..

[26] Nejadghaderi SA, Nazemalhosseini-Mojarad E, Asadzadeh Aghdaei H (2021). Fecal microbiota transplantation for COVID-19; a potential emerging treatment strategy. Med. Hypotheses.

[27] Metta V (2021). Gastrointestinal dysfunction in Parkinson’s disease: Molecular pathology and implications of gut microbiome, probiotics, and fecal microbiota transplantation. J. Neurol..

[28] Bakken JS (2011). Treating *Clostridium difficile* infection with fecal microbiota transplantation. Clin. Gastroenterol. Hepatol..

[29] Li T (2022). Neuroprotective effects of bifidobacterium breve CCFM1067 in MPTP-induced mouse models of Parkinson’s disease. Nutrients.

[30] Liberatore GT (1999). Inducible nitric oxide synthase stimulates dopaminergic neurodegeneration in the MPTP model of Parkinson disease. Nat. Med..

[31] Li H (2020). Probiotic mixture of *Lactobacillus plantarum* strains improves lipid metabolism and gut microbiota structure in high fat diet-fed mice. Front. Microbiol..

[32] Aktas B (2015). The effect of *Lactobacillus casei* 32G on the mouse cecum microbiota and innate immune response is dose and time dependent. PLoS ONE.

[33] Bonfrate L (2020). Effects of *Bifidobacterium longum* BB536 and *Lactobacillus rhamnosus* HN001 in IBS patients. Eur. J. Clin. Invest..

[34] Gao Y (2021). *Lactobacillus plantarum* Y44 alleviates oxidative stress by regulating gut microbiota and colonic barrier function in Balb/C mice with subcutaneous d-galactose injection. Food Funct..

[35] Mazziotta C, Tognon M, Martini F, Torreggiani E, Rotondo JC (2023). Probiotics mechanism of action on immune cells and beneficial effects on human health. Cells.

[36] Tillisch K (2013). Consumption of fermented milk product with probiotic modulates brain activity. Gastroenterology.

[37] Tamtaji OR (2018). Clinical and metabolic response to probiotic administration in people with Parkinson’s disease: A randomized, double-blind, placebo-controlled trial. Clin. Nutr..

[38] Tan AH (2021). Probiotics for constipation in Parkinson disease: A randomized placebo-controlled study. Neurology.

[39] Ibrahim A (2020). Multi-strain probiotics (Hexbio) containing MCP BCMC strains improved constipation and gut motility in Parkinson’s disease: A randomised controlled trial. PLoS ONE.

[40] Barichella M, Bolliri C, Cassani E, Iorio L, Pusani C, Pinelli G, Privitera G, Cesari I, Faierman SA, Caccialanza R, Pezzoli G, Cereda EPC (2016). Probiotics and prebiotic fiber for constipation associated with Parkinson disease: An RCT. Neurology.

[41] Pan S (2022). Probiotic *Pediococcus pentosaceus* ameliorates MPTP-induced oxidative stress via regulating the gut microbiota–gut–brain axis. Front. Cell. Infect. Microbiol..

[42] Sun J (2021). Probiotic *Clostridium butyricuma* ameliorated motor deficits in a mouse model of Parkinson’s disease via gut microbiota-GLP-1 pathway. Brain. Behav. Immun..

[43] Dolanbay S, Aslim B (2022). Comparison of the anti-carcinogenic effects of some probiotic bacteria and their postbiotics on. J. Appl. Biol. Sci..

[44] Tukenmez U, Aktas B, Aslim B, Yavuz S (2019). The relationship between the structural characteristics of lactobacilli-EPS and its ability to induce apoptosis in colon cancer cells in vitro. Sci. Rep..

[45] Mendi A, Aslim B (2022). Exopolysaccharide of *Lactobacillus rhamnosus* E9 strain improves dental pulp mesenchymal stem cell proliferation, osteogenic differentiation, and cellular collagen production. Braz. Arch. Biol. Technol..

[46] Sánchez B, Bressollier P, Urdaci MC (2008). Exported proteins in probiotic bacteria: Adhesion to intestinal surfaces, host immunomodulation and molecular cross-talking with the host. FEMS Immunol. Med. Microbiol..

[47] Ciszek-Lenda M (2011). Strain specific immunostimulatory potential of lactobacilli-derived exopolysaccharides. Cent. Eur. J. Immunol..

[48] Nikolic M (2012). Characterisation of the exopolysaccharide (EPS)-producing *Lactobacillus paraplantarum* BGCG11 and its non-EPS producing derivative strains as potential probiotics. Int. J. Food Microbiol..

[49] Sungur T, Aslim B, Karaaslan C, Aktas B (2017). Impact of Exopolysaccharides (EPSs) of *Lactobacillus gasseri* strains isolated from human vagina on cervical tumor cells (HeLa). Anaerobe.

[50] Song J (2016). Investigation of the antidepressant effects of exopolysaccharides obtained from *Marasmius androsaceus* fermentation in a mouse model. Mol. Med. Rep..

[51] Sirin S, Aslim B (2020). Characterization of lactic acid bacteria derived exopolysaccharides for use as a defined neuroprotective agent against amyloid beta1–42-induced apoptosis in SH-SY5Y cells. Sci. Rep..

[52] Sirin S, Aslim B (2021). Protective effect of exopolysaccharides from lactic acid bacteria against amyloid beta1-42induced oxidative stress in SH-SY5Y cells: Involvement of the AKT, MAPK, and NF-κB signaling pathway. Process Biochem..

[53] Yildiz GG, Öztürk M, Aslim B (2011). Identification of Lactobacillus strains from breast-fed infant and investigation of their cholesterol-reducing effects. World J. Microbiol. Biotechnol..

[54] du Sert NP (2020). The arrive guidelines 2.0: Updated guidelines for reporting animal research. PLoS Biol..

[55] Haque ME (2021). The neuroprotective effects of GPR4 inhibition through the attenuation of caspase mediated apoptotic cell death in an MPTP induced mouse model of Parkinson’s disease. Int. J. Mol. Sci..

[56] He R, Huang W, Huang Y, Xu M, Song P (2018). Cdk5 inhibitory peptide prevents loss of dopaminergic neurons and alleviates behavioral changes in an MPTP induced Parkinson’s disease mouse model. Front. Aging Neurosci..

[57] Rodriguez A (2018). ToxTrac: A fast and robust software for tracking organisms. Methods Ecol. Evol..

[58] Bhattacharjee N, Paul R, Giri A, Borah A (2016). Chronic exposure of homocysteine in mice contributes to dopamine loss by enhancing oxidative stress in nigrostriatum and produces behavioral phenotypes of Parkinson’s disease. Biochem. Biophys. Rep..

[59] Feng P (2018). Two novel dual GLP-1/GIP receptor agonists are neuroprotective in the MPTP mouse model of Parkinson’s disease. Neuropharmacology.

[60] Aktas B (2023). Gut microbial alteration in MPTP mouse model of Parkinson disease is administration regimen dependent. Cell. Mol. Neurobiol..

[61] Dutta M (2016). Metabolomics reveals altered lipid metabolism in a mouse model of endometriosis. J. Proteome Res..

[62] Johnson ECB (2018). Deep proteomic network analysis of Alzheimer’s disease brain reveals alterations in RNA binding proteins and RNA splicing associated with disease. Mol. Neurodegener..

[63] Liao JF (2020). *Lactobacillus plantarum* PS128 alleviates neurodegenerative progression in 1-methyl-4-phenyl-1,2,3,6-tetrahydropyridine-induced mouse models of Parkinson’s disease. Brain. Behav. Immun..

[64] Du Y (2001). Minocycline prevents nigrostriatal dopaminergic neurodegeneration in the MPTP model of Parkinson’s disease. Proc. Natl. Acad. Sci..

[65] Dray S, Dufour A-B (2007). Journal of statistical software. J. Stat. Softw..

[66] Chessel D, Dufour AB, Thioulouse J (2004). The ade4 package-I: One-table methods. R News.

[67] The R Core Team. *R : A Language and Environment for Statistical Computing*. **1** (2013).

[68] de Cárcer DA (2011). Numerical ecology validates a biogeographical distribution and gender-based effect on mucosa-associated bacteria along the human colon. ISME J..

[69] Wang L (2021). Neuroprotective effect of *Lactobacillus plantarum* DP189 on MPTP-induced Parkinson’s disease model mice. J. Funct. Foods.

[70] Chu C (2023). *Lactobacillus plantarum* CCFM405 against rotenone-induced Parkinson’s disease mice via regulating gut microbiota and branched-chain amino acids biosynthesis. Nutrients.

[71] Wu H (2022). Neuroprotective effects of an engineered *Escherichia coli* Nissle 1917 on Parkinson’s disease in mice by delivering GLP-1 and modulating gut microbiota. Bioeng. Transl. Med..

[72] Xie C, Prasad AA (2020). Probiotics treatment improves hippocampal dependent cognition in a rodent model of Parkinson’s disease. Microorganisms.

[73] Huang D (2018). Long-term changes in the nigrostriatal pathway in the MPTP mouse model of Parkinson’s disease. Neuroscience.

[74] Kuroiwa H, Yokoyama H, Kimoto H, Kato H, Araki T (2010). Biochemical alterations of the striatum in an MPTP-treated mouse model of Parkinson’s disease. Metab. Brain Dis..

[75] Schwarting RKW, Sedelis M, Hofele K, Auburger GW, Huston JP (1999). Strain-dependent recovery of open-field behavior and striatal dopamine deficiency in the mouse MPTP model of Parkinson’s disease. Neurotox. Res..

[76] Liu J (2014). Neuroprotective effects of Jitai tablet, a traditional Chinese medicine, on the MPTP-induced acute model of Parkinson’s disease: Involvement of the dopamine system. Evid. Based Complement. Altern. Med..

[77] Jeon H, Bae CH, Lee Y, Kim HY, Kim S (2021). Korean red ginseng suppresses 1-methyl-4-phenyl-1,2,3,6-tetrahydropyridine-induced inflammation in the substantia nigra and colon. Brain. Behav. Immun..

[78] Lu Y (2018). Metabolic disturbances in the striatum and substantia nigra in the onset and progression of MPTP-induced Parkinsonism model. Front. Neurosci..

[79] Al Sweidi S, Morissette M, Rouillard C, Di Paolo T (2013). Estrogen receptors and lesion-induced response of striatal dopamine receptors. Neuroscience.

[80] Pain S (2013). Toxicity of MPTP on neurotransmission in three mouse models of Parkinson’s disease. Exp. Toxicol. Pathol..

[81] Rico AJ (2017). Neurochemical evidence supporting dopamine D1–D2 receptor heteromers in the striatum of the long-tailed macaque: Changes following dopaminergic manipulation. Brain Struct. Funct..

[82] Smith TS, Trimmer PA, Khan SM, Tinklepaugh DL, Bennett JP (1997). Mitochondrial toxins in models of neurodegenerative diseases. II: Elevated zif268 transcription and independent temporal regulation of striatal D1 and D2 receptor mRNAs and D1 and D2 receptor-binding sites in C57BL/6 mice during MPTP treatment. Brain Res..

[83] Tanji H, Araki T, Nagasawa H, Itoyama Y (1999). Differential vulnerability of dopamine receptors in the mouse brain treated with MPTP. Brain Res..

[84] Yang P (2021). Dopamine D1 + D3 receptor density may correlate with Parkinson disease clinical features. Ann. Clin. Transl. Neurol..

[85] Calabrese V (2018). Aging and Parkinson’s disease: Inflammaging, neuroinflammation and biological remodeling as key factors in pathogenesis. Free Radic. Biol. Med..

[86] Dias V, Junn E, Mouradian MM (2013). The role of oxidative stress in Parkinson’s disease. J. Parkinsons. Dis..

[87] Wang L (2022). *Lactobacillus plantarum* DP189 reduces α-SYN aggravation in MPTP-induced Parkinson’s disease mice via regulating oxidative damage, inflammation, and gut microbiota disorder. J. Agric. Food Chem..

[88] van Rensburg ZJ, Abrahams S, Bardien S, Kenyon C (2021). Toxic feedback loop involving iron, reactive oxygen species, α-synuclein and neuromelanin in Parkinson’s disease and intervention with turmeric. Mol. Neurobiol..

[89] Clairembault T (2014). Enteric GFAP expression and phosphorylation in Parkinson’s disease. J. Neurochem..

[90] Devos D (2013). Colonic inflammation in Parkinson’s disease. Neurobiol. Dis..

[91] Forsyth CB (2011). Increased intestinal permeability correlates with sigmoid mucosa alpha-synuclein staining and endotoxin exposure markers in early Parkinson’s disease. PLoS ONE.

[92] Benvenuti L (2020). Enteric glia at the crossroads between intestinal immune system and epithelial barrier: Implications for Parkinson disease. Int. J. Mol. Sci..

[93] Chu H, Mazmanian SK (2013). Innate immune recognition of the microbiota promotes host-microbial symbiosis. Nat. Immunol..

[94] Abreu MT (2010). Toll-like receptor signalling in the intestinal epithelium: How bacterial recognition shapes intestinal function. Nat. Rev. Immunol..

[95] Aktas B, De Wolfe TJ, Safdar N, Darien BJ, Steele JL (2016). The impact of *Lactobacillus casei* on the composition of the cecal microbiota and innate immune system is strain specific. PLoS ONE.

[96] Akira S, Takeda K (2004). Toll-like receptor signalling. Nat. Rev. Immunol..

[97] Kekkonen RA (2008). Probiotic intervention has strain-specific anti-inflammatory effects in healthy adults. World J. Gastroenterol..

[98] Christensen HR, Frokiaer H, Pestka JJ, Alerts E (2002). Lactobacilli differentially modulate expression of cytokines and maturation surface markers in murine dendritic cells. J. Immunol..

[99] Schuijt TJ, van der Poll T, de Vos WM, Wiersinga WJ (2013). The intestinal microbiota and host immune interactions in the critically ill. Trends Microbiol..

[100] Lacy P, Stow JL (2011). Cytokine release from innate immune cells: Association with diverse membrane trafficking pathways. Blood.

[101] Kirschning CJ, Schuman RR (2002). Toll-Like Receptor Family Members and Their Ligands.

[102] *Fundamental Immunology*. (Lippincott Williams & Wilkins, 2003).

[103] Houser MC, Tansey MG (2017). The gut-brain axis: Is intestinal inflammation a silent driver of Parkinson’s disease pathogenesis?. NPJ Park. Dis..

[104] Ostaff MJ, Stange EF, Wehkamp J (2013). Antimicrobial peptides and gut microbiota in homeostasis and pathology. EMBO Mol. Med..

[105] Gill SR (2006). Metagenomic analysis of the human distal gut microbiome. Science.

[106] Parra I (2023). Neuroprotective and immunomodulatory effects of probiotics in a rat model of Parkinson’s disease. Neurotox. Res..

[107] Sun H (2022). Probiotics synergized with conventional regimen in managing Parkinson’s disease. NPJ Park. Dis..

[108] Nguyen TLA, Vieira-Silva S, Liston A, Raes J (2015). How informative is the mouse for human gut microbiota research?. Dis. Model. Mech..

[109] Sokol H (2009). Low counts of *Faecalibacterium prausnitzii* in colitis microbiota. Inflamm. Bowel Dis..

[110] Keshavarzian A (2015). Colonic bacterial composition in Parkinson’s disease. Mov. Disord..

[111] Pereira FC (2020). Rational design of a microbial consortium of mucosal sugar utilizers reduces *Clostridiodes difficile* colonization. Nat. Commun..

[112] Liu X (2021). Intragastric administration of casein leads to nigrostriatal disease progressed accompanied with persistent nigrostriatal—intestinal inflammation activited and intestinal microbiota—metabolic disorders induced in MPTP mouse model of Parkinson’s disease. Neurochem. Res..

[113] Takahashi K (2022). Altered gut microbiota in Parkinson’s disease patients with motor complications. Park. Relat. Disord..

[114] Ratto D (2022). The many ages of microbiome–gut–brain axis. Nutrients.

[115] Perez-Pardo P (2019). Role of TLR4 in the gut-brain axis in Parkinson’s disease: A translational study from men to mice. Gut.

[116] Zheng J (2020). A taxonomic note on the genus Lactobacillus: Description of 23 novel genera, emended description of the genus *Lactobacillus beijerinck* 1901, and union of Lactobacillaceae and Leuconostocaceae. Int. J. Syst. Evol. Microbiol..

[117] NIH. *Probiotics: Fact Sheet for Health Professionals*. (2022). https://ods.od.nih.gov/factsheets/Probiotics-HealthProfessional. Accessed 10 June 2023.

[118] Bravo JA (2011). Ingestion of Lactobacillus strain regulates emotional behavior and central GABA receptor expression in a mouse via the vagus nerve. Proc. Natl. Acad. Sci..

[119] Liu X (2022). Polymannuronic acid prebiotic plus *Lacticaseibacillus rhamnosus* GG probiotic as a novel synbiotic promoted their separate neuroprotection against Parkinson’s disease. Food Res. Int..

[120] Quan Y, Zhang KX, Zhang HY (2023). The gut microbiota links disease to human genome evolution. Trends Genet..

[121] Nowak JM, Kopczyński M, Friedman A, Koziorowski D, Figura M (2022). Microbiota dysbiosis in Parkinson disease: In search of a biomarker. Biomedicines.

[122] Zhang J-C (2017). Blockade of interleukin-6 receptor in the periphery promotes rapid and sustained antidepressant actions: A possible role of gut–microbiota–brain axis. Transl. Psychiatry.

[123] Peng L, Li ZR, Green RS, Holzman IR, Lin J (2009). Butyrate enhances the intestinal barrier by facilitating tight junction assembly via activation of AMP-activated protein kinase in Caco-2 cell monolayers. J. Nutr..

[124] Kong Q (2021). The autistic-like behaviors development during weaning and sexual maturation in VPA-induced autistic-like rats is accompanied by gut microbiota dysbiosis. PeerJ.

