# Supplementary material for: A neurotherapeutic approach with Lacticaseibacillus rhamnosus E9 on gut microbiota and intestinal barrier in MPTP-induced mouse model of Parkinson’s disease
Source: Sci Rep. 2024 Jul 4;14:15460. doi: 10.1038/s41598-024-65061-w (PMC11224381; doi:10.1038/s41598-024-65061-w)
Supplement: Supplementary file 1 — Supplementary Information. [file 41598_2024_65061_MOESM1_ESM.pdf]

A Neurotherapeutic Approach with *Lacticaseibacillus rhamnosus* E9 on Gut Microbiota and Intestinal Barrier in MPTP-induced Mouse Model of Parkinson's Disease

Busra Aktas[1*], Belma Aslim[2], Deniz Ates Ozdemir[3]

[1] Department of Molecular Biology and Genetics, Burdur Mehmet Akif Ersoy University, Burdur, 15200 Turkey

[2] Department of Biology, Faculty of Science, Gazi University, Ankara, 06500 Turkey

[3] Department of Pathology, Faculty of Medicine, Hacettepe University, Ankara, 06230 Turkey

[*] Corresponding author: aktas@uwalumni.com

Busra Aktas ORCID ID: 0000-0001-9863-683X

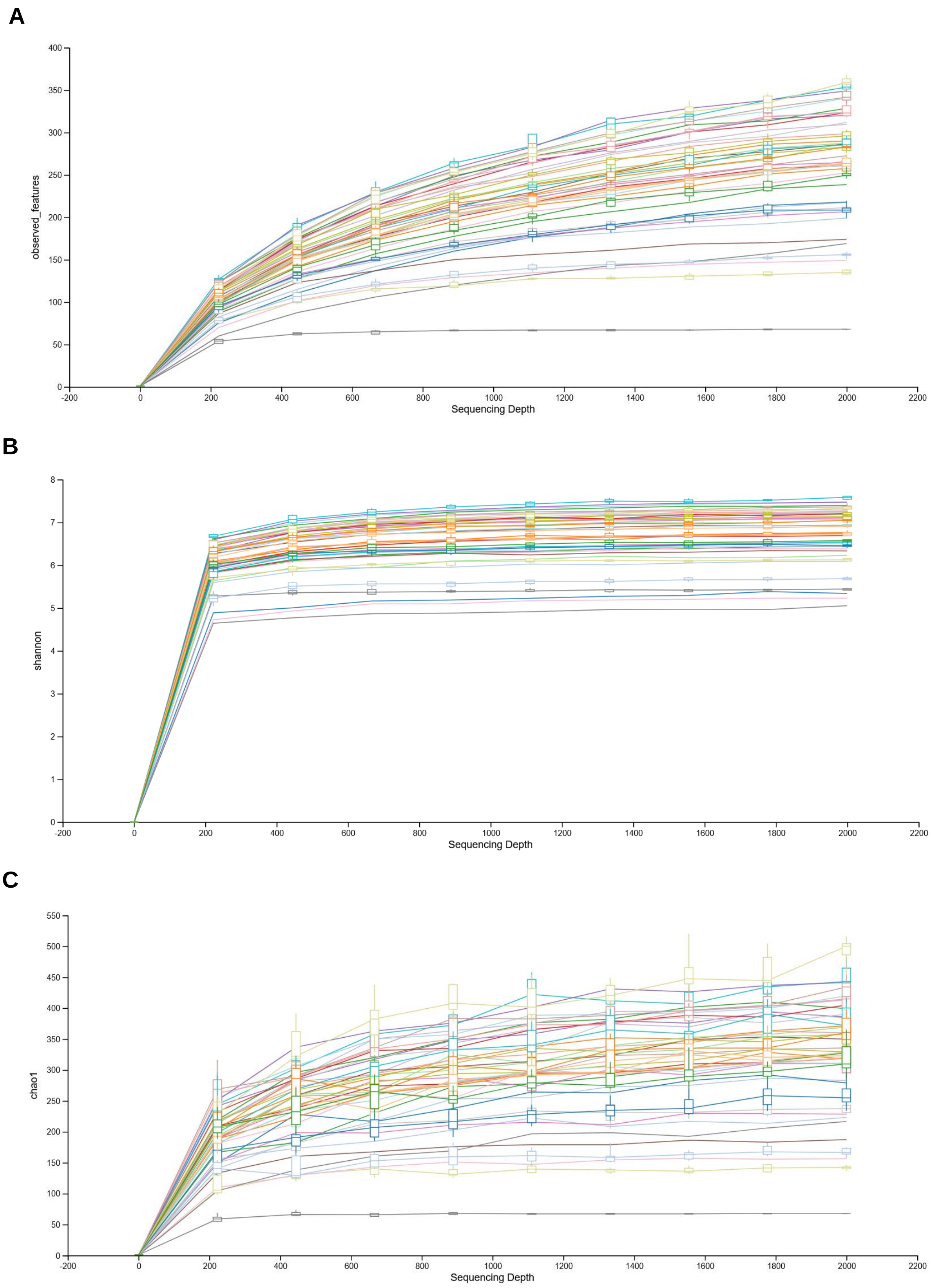
A
observed_features
Sequencing Depth
B
shannon
Sequencing Depth
C
chao1
Sequencing Depth

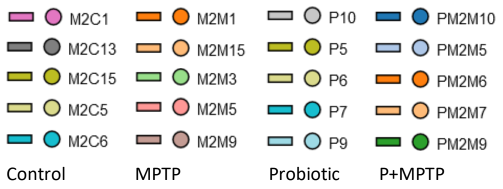

Figure S1. Alpha rarefacton plots based on Observed features (A), Shannon (B), and Chao1index (C).

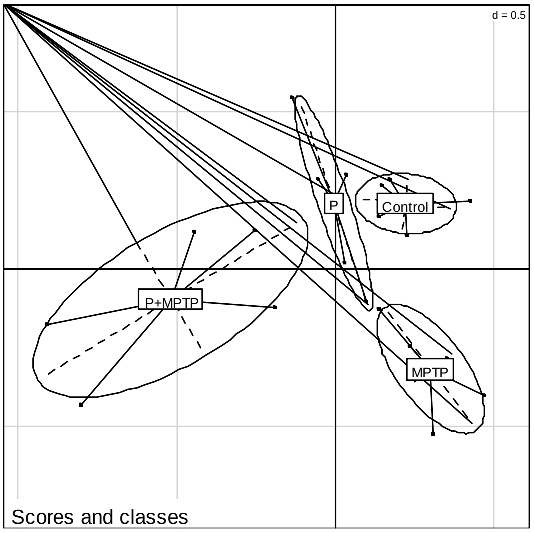

Figure S2. The microbiota composition of mouse cecum digesta at the genus level was clustered by MPTP administration regimen using between group analysis (BGA).

Table S1. Primers used in qPCR analysis.

Gene	Forward Primer	Reverse Primer	References
D1	5'- ATGGCTCCTAACACTTCTACCA -3'	5'- GGGTATTCCCTAAGAGAGTGGAC -3'	1
D2	5'- ACCTGTCCTGGTACGATGATG -3'	5'- GCATGGCATAGTAGTTGTAGTGG -3'	1
DAT	5'- GCCCCTGCTTCCTTCTGTATGT -3'	5'- CACTAGCTGGCGGTCTTTCTCA -3'	2
β-actin	5'-CTGACAGACTACCTCATGAAGATCC-3'	5'-AGTCTAGAGCAACATAGCACAGCTT-3'	3

[1] Y. Li, J. Rong, H. Zhong, M. Liang, C. Zhu, F. Chang, and R. Zhou, Mol. Neurobiol. **58**, 317 (2021).
[2] E.R. Mingazov, G.R. Khakimova, E.A. Kozina, A.E. Medvedev, O.A. Buneeva, A.S. Bazyan, and M. V. Ugrumov, Mol. Neurobiol. **55**, 2991 (2018).
[3] T.R. Bagalkot, H.M. Jin, V. V. Prabhu, S.S. Muna, Y. Cui, B.K. Yadav, H.J. Chae, and Y.C. Chung, Neuroscience **311**, 444 (2015).

Table S2. Bacterial phyla detected in cecum content of mice administered MPTP and/or Probiotic.

Taxon	Percentage (mean ± SE)[ab]			
	Control	MPTP	Probiotic	P+MPTP
Firmicutes	48.1 ± 3.7	**41.6 ± 2.6**	47.1 ± 4.9	45.2 ± 5.5
Bacteroidota	37.9 ± 7.7	**48.4 ± 1.2**	42.6 ± 2.7	38.5 ± 7.4
Desulfobacterota	5.4 ± 4.3	3.3 ± 1.2	**2.4 ± 0.9**	**2.0 ± 0.9**
Patescibacteria	3.7 ± 1.5	**1.3 ± 1.1**	2.8 ± 1.4	**0.7 ± 1.1**
Campylobacterota	2.4 ± 2.4	**0.7 ± 0.4**	**0.6 ± 0.5**	1.3 ± 0.9
Proteobacteria	1.8 ± 1.3	1.8 ± 1.3	**0.8 ± 0.5**	5.2 ± 7.3
Actinobacteriota	0.4 ± 0.3	0.2 ± 0.2	0.9 ± 1.2	0.5 ± 0.5
Cyanobacteria	0.3 ± 0.3	0.1 ± 0.1	0.4 ± 0.7	0.5 ± 0.3
Parabasalia	0.1 ± 0.1	0.0 ± 0.0	0.3 ± 0.3	0.2 ± 0.2
Unassigned; Unknown_1	0.1 ± 0.1	0.1 ± 0.1	0.0 ± 0.1	0.0 ± 0.0
Eukaryota; Unknown_2	0.0 ± 0.0	0.0 ± 0.0	0.0 ± 0.0	0.0 ± 0.0
Bacteria; Unknown_3	0.0 ± 0.0	0.0 ± 0.0	0.0 ± 0.0	**0.0 ± 0.0**
Verrucomicrobiota	0.0 ± 0.0	**2.2 ± 2.2**	1.9 ± 3.4	5.8 ± 9.5
Spirochaetota	0.0 ± 0.0	0.2 ± 0.4	0.1 ± 0.2	**0.0 ± 0.0**
Fusobacteriota	0.0 ± 0.0	0.0 ± 0.0	0.0 ± 0.0	**0.0 ± 0.0**
Deferribacterota	0.0 ± 0.0	0.0 ± 0.0	0.0 ± 0.0	**0.0 ± 0.0**

[a] The detection limit was 0.000021 and this value was used to calculate the p-value.
[b] Phyla that differ from control within each group are shown in bold ($p \leq 0.05$). The statistical difference was examined using the Monte-Carlo test.

Table S3. Bacterial genera detected[a] in cecum content of mice administered MPTP and/or Probiotic.

Taxon	Percentage (mean ± SE)[bc]			
	Control	MPTP	Probiotic	PMPTP
Muribaculaceae	24.6 ± 9.1	29.8 ± 2.8	24.4 ± 2.3	21.4 ± 8.6
Lachnospiraceae_NK4A136_group	16.9 ± 1.2	16.0 ± 2.6	16.0 ± 3.1	**10.0 ± 3.6**
Lachnospiraceae; Unknown_1	4.6 ± 2.8	3.7 ± 0.8	5.5 ± 1.4	5.4 ± 2.1
Candidatus_Saccharimonas	3.7 ± 1.5	**1.3 ± 1.1**	2.8 ± 1.4	**0.7 ± 1.1**
Oscillospiraceae; Unknown_2	3.5 ± 2.8	2.3 ± 0.6	1.5 ± 0.9	1.7 ± 0.6
Colidextribacter	3.1 ± 1.4	2.6 ± 0.2	**1.8 ± 0.9**	**1.8 ± 0.6**
Oscillibacter	3.0 ± 2.0	1.8 ± 1.1	**1.5 ± 0.8**	2.5 ± 1.0
Desulfovibrionaceae; Uncultured_1	3.0 ± 4.6	2.5 ± 1.5	0.5 ± 0.7	0.8 ± 1.0
Alloprevotella	3.0 ± 3.5	4.5 ± 2.6	1.8 ± 1.3	1.3 ± 2.2
Prevotellaceae_NK3B31_group	2.6 ± 2.3	1.7 ± 1.4	5.0 ± 5.3	**6.3 ± 3.3**
Helicobacter	2.4 ± 2.4	**0.7 ± 0.4**	**0.6 ± 0.5**	1.3 ± 0.9
Bacteroides	2.3 ± 1.2	1.8 ± 1.1	3.3 ± 2.4	3.3 ± 2.1
Desulfovibrio	2.0 ± 1.5	**0.8 ± 0.5**	1.7 ± 1.1	1.0 ± 0.5
Prevotellaceae_UCG-001	1.9 ± 1.3	1.8 ± 0.5	2.9 ± 2.2	1.7 ± 1.5
Oscillospiraceae; Uncultured_3	1.8 ± 0.9	2.4 ± 1.1	1.6 ± 0.9	1.6 ± 0.6
Lachnospiraceae; Uncultured_5	1.7 ± 0.7	1.3 ± 0.5	1.4 ± 1.0	2.1 ± 0.8
Lachnospiraceae_UCG-001	1.6 ± 0.6	1.2 ± 1.0	**3.3 ± 2.8**	**4.4 ± 2.9**
Muribaculum	1.3 ± 0.8	1.4 ± 1.0	2.0 ± 1.0	1.0 ± 0.8
Anaerotruncus	1.0 ± 1.1	**0.2 ± 0.2**	0.4 ± 0.2	0.6 ± 0.5
Parasutterella	1.0 ± 0.8	1.2 ± 1.2	0.4 ± 0.4	0.7 ± 0.9
Peptococcaceae; Uncultured_2	0.9 ± 0.3	0.7 ± 0.3	**0.5 ± 0.3**	**0.4 ± 0.3**
Ruminococcaceae; Uncultured_4	0.8 ± 0.5	0.8 ± 0.9	0.7 ± 0.5	**0.4 ± 0.3**
Alistipes	0.8 ± 0.3	1.1 ± 0.4	1.0 ± 0.5	1.2 ± 0.8
Clostridia_UCG-014	0.7 ± 0.5	**0.3 ± 0.2**	0.7 ± 0.2	0.6 ± 0.7
Intestinimonas	0.7 ± 0.5	0.9 ± 0.5	0.7 ± 0.8	0.4 ± 0.5
Ruminococcus	0.7 ± 1.2	0.5 ± 0.3	0.4 ± 0.3	1.7 ± 2.5
Lactobacillus	0.7 ± 0.6	**0.2 ± 0.1**	1.1 ± 1.0	0.6 ± 0.7
Ligilactobacillus	0.6 ± 0.8	**0.1 ± 0.1**	1.5 ± 2.3	0.6 ± 0.5
Roseburia	0.5 ± 0.4	0.6 ± 0.4	1.1 ± 0.9	1.3 ± 1.4
Butyricicoccus	0.4 ± 0.3	**0.1 ± 0.1**	0.2 ± 0.2	0.5 ± 0.5
Prevotellaceae; Unknown_3	0.4 ± 0.6	**1.4 ± 1.0**	0.4 ± 0.6	**0.0 ± 0.0**
Monoglobus	0.4 ± 0.4	0.2 ± 0.2	0.2 ± 0.1	0.6 ± 0.8
Incertae_Sedis	0.4 ± 0.5	0.2 ± 0.2	0.5 ± 0.4	1.1 ± 1.3
Escherichia-Shigella	0.4 ± 0.7	0.3 ± 0.6	0.0 ± 0.0	3.5 ± 7.8
Eubacterium_coprostanoligenes_group	0.3 ± 0.3	0.7 ± 0.9	0.5 ± 0.6	**1.1 ± 1.1**
Gastranaerophilales	0.3 ± 0.3	0.1 ± 0.1	0.4 ± 0.7	0.5 ± 0.3
Lachnoclostridium	0.2 ± 0.3	0.2 ± 0.1	0.4 ± 0.3	0.5 ± 0.4
Prevotellaceae_Ga6A1_group	0.2 ± 0.2	**4.1 ± 3.4**	1.3 ± 2.0	0.5 ± 1.2
uncultured; Uncultured_6	0.1 ± 0.2	0.0 ± 0.0	0.0 ± 0.0	0.9 ± 2.0
Allobaculum	0.0 ± 0.0	0.5 ± 1.0	0.0 ± 0.1	0.1 ± 0.1
Akkermansia	**BQL**	**2.2 ± 2.2**	1.9 ± 3.4	5.8 ± 9.5
Lacticaseibacillus	**BQL**	**BQL**	**0.6 ± 0.8**	**0.2 ± 0.3**

[a] Only genera that were present at ≥1% in a sample are included in this table.
[b] The detection limit was 0.000021 and this value was used to calculate the p-value.
[c] Phyla that differ from control within each group are shown in bold ($p \leq 0.05$). The statistical difference was examined using the Monte-Carlo test.
BQL: Below quantifiable limit.
